# Efficacy and Safety of Ondansetron in Schizophrenia: A Systematic Review and Meta-Analysis of Randomized Controlled Trials

**DOI:** 10.1192/bjo.2025.10133

**Published:** 2025-06-20

**Authors:** Omar Kassar, Osama Hassan, Omar Shaheen, Abdullah Selim, Moaz Elsayed Abouelmagd

**Affiliations:** ^1^Faculty of Medicine, Alexandria University, Alexandria, Egypt; ^2^Medical Research Group of Egypt, Neida Academy, Arlington, Massachusetts, USA; ^3^Central and North West London NHS Foundation Trust, London, United Kingdom; ^4^Faculty of Medicine, Zagazig University, Zagazig, Egypt; ^5^Faculty of Medicine, Cairo University, Cairo, Egypt

## Abstract

**Aims:** It has been shown that 5-hydroxytryptamine3 (5-HT3) receptors are involved in the pathogenesis of schizophrenia. This systematic review and meta-analysis of randomized clinical trials (RCTs) evaluates the efficacy and safety of ondansetron, a potent 5-HT3 receptor antagonist, as adjunctive treatment for the management of schizophrenia, especially the negative symptoms and cognitive deficits.

**Methods:** A comprehensive search of electronic databases, including PubMed, Scopus, Cochrane, and Web of Science, was performed in October 2024. We included only randomized controlled trials (RCTs), and their data were extracted and analysed using RevMan 5.4 software. The primary outcome was the PANSS (Positive and Negative Syndrome Scale) negative subscale.

**Results:** Eight RCTs involving 533 patients were included in the study. Ondansetron showed a statistically significant improvement in PANSS negative subscale at 12 weeks [pooled as mean difference, MD=−2.96, 95% CI [−4.69, −1.24], p=0.00007] and in general psychopathology scale [MD= −2.71, 95% CI [−3.52, −1.90]] compared with placebo. However, ondansetron and placebo did not differ in reduction of PANSS positive subscale [MD= 0.1, 95% CI [−1.19, 1.38], p=0.88], and depression scale (SMD= 0.71, 95% [−0.35, 1.77], p=0.19). Ondansetron showed no significant difference regarding tardive dyskinesia between the two groups. However, constipation was significant in the ondansetron group over placebo.

**Conclusion:** The study’s findings support the use of ondansetron as adjuvant therapy in the management of schizophrenia, particularly the negative symptoms and cognitive deficits.

